# Quantifying the impact of adherence to screening strategies on colorectal cancer incidence and mortality

**DOI:** 10.1002/cam4.2735

**Published:** 2019-11-28

**Authors:** Elvira D’Andrea, Dennis J. Ahnen, Daniel A. Sussman, Mehdi Najafzadeh

**Affiliations:** ^1^ Division of Pharmacoepidemiology and Pharmacoeconomics Department of Medicine Brigham and Women’s Hospital Harvard Medical School Boston Massachusetts; ^2^ School of Medicine and Gastroenterology of the Rockies University of Colorado Denver Colorado; ^3^ Division of Gastroenterology Department of Medicine University of Miami Miller School of Medicine Miami Florida

**Keywords:** adherence, colorectal cancer screening, compliance, simulation modeling

## Abstract

Current recommendations of The US Preventive Services Task Force (USPSTF) on colorectal cancer (CRC) screening strategies are based on models that assume 100% adherence. Since adherence can have a large effect on screening outcomes, we aimed to compare the effectiveness of CRC screening strategies under reported adherence rates at the population level. We developed and validated a microsimulation model to assess the effectiveness of colonoscopy (COL), flexible sigmoidoscopy (FS), high‐sensitivity guaiac fecal occult blood‐test (HS‐gFOBT), fecal immunochemical test (FIT), multitarget stool DNA test (FIT‐DNA), computed tomography colonography (CTC), and methylated SEPT9 DNA test (SEPT9) in terms of CRC incidence and mortality, incremental life years gained (LYG), number of colonoscopies, and adverse events for men and women 50 years or older over their lifetime. We assessed outcomes under 100% adherence rates and reported adherence rates. We also performed sensitivity analyses to evaluate the impact of varying adherence levels on CRC outcomes. Assuming 100% adherence, FIT‐DNA, FIT, HS‐gFOBT, and SEPT9 averted 42‐45 CRC cases and 25‐26 CRC deaths, COL 46 cases and 26 deaths, CTC 39 cases and 23 deaths, FS 32 cases and 19 deaths per 1000 individuals. Assuming reported adherence, SEPT9 averted 37 CRC cases and 23 CRC deaths, COL 34 cases and 20 deaths, FIT‐DNA, FIT, CTC and HS‐gFOBT 16‐25 cases and 10‐16 deaths per 1000 individuals. LYG reflected the effectiveness of each strategy in reducing CRC cases and deaths. Adverse events were more common for COL (3.7 per 1000 screened) and annual SEPT9 (3.4 per 1000 screened), and proportional to the number of colonoscopies. Among the screening strategies recommended by USPSTF, colonoscopy results in the largest benefit when we account for adherence. Adherence rates higher than 65%‐70% would be required for any stool or blood‐based screening modality to match the benefits of colonoscopy.

## Graphical abstract

Although adherence to colorectal cancer screening has a substantial impact on clinical outcomes, estimating the magnitude of this impact has been challenging. This study provides a quantitative analysis of the extent to which differences in adherence rates affect clinical outcomes of different screening strategies. We found that higher adherence can lead to meaningful benefits when compared to strategies with better one‐time sensitivity and/or specificity, but lower adherence rates. This is relevant for optimizing screening strategies when considering other factors in addition to test performance, such as access to screening and patient behavior.

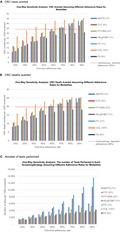

## 1 INTRODUCTION

The US Preventive Services Task Force (USPSTF) strongly recommends (A grade) screening for colorectal cancer (CRC), at regular intervals, beginning at age 50 years and continuing until age 75 years.^1^ Although there is high certainty for substantial net benefit of CRC screening, there is no agreement on the superiority of a single screening strategy.^1^, ^2^ In fact, factors other than the accuracy of the tests (eg, compliance with the test, screening intervals, prevalence of neoplasia in the screened population, and family history of CRC) can affect the overall benefit of a specific strategy in clinical practice.^2^, ^3^

There are limited observational data on ongoing CRC screening programs to perform a full assessment on the effectiveness of alternative screening strategies.^4^ Thus, simulation models play a central role for evaluating the harms and benefits among different strategies.^1^ Current recommendations of the USPSTF for CRC screening are partially based on the results of three Cancer Intervention and Surveillance Modeling Network (CISNET) simulation models.^5^ The most recent analyses published by the USPSTF aimed to assess the effectiveness of CRC screening strategies assuming full (100%) adherence to screening. Under the assumption of full adherence, findings on the effectiveness of alternative CRC screening strategies are primarily driven by the performance characteristics of screening tests and assume only the standpoint of individuals fully compliant to screening recommendations. Nevertheless, full adherence to screening is rarely observed at the population level, and even for tests with very high diagnostic accuracy, such as colonoscopy, the benefit of screening could be muted by a suboptimal uptake.^2^

In this study, we developed and validated a microsimulation model to simulate colorectal cancer incidence and mortality based on observed data. We then used the validated model to assess clinical benefits, harms, and burden of testing across all available strategies for CRC screening, under both full and reported adherence. Adherence to screening has several dimensions and may be influenced by predisposing, enabling or *need* factors such as age, race, education, economic status and insurance coverage, family history of cancer or current colorectal disease, etc^6^. In our study, reported adherence estimates were extracted from peer‐reviewed literature, which accounted for most of these factors. We used the adherence rates “reported” in the literature for the base case scenario and performed several sensitivity analyses, across a range of adherence levels, for each screening strategy.

## 2 METHODS

### 2.1 Simulation model

**Figure 1:**
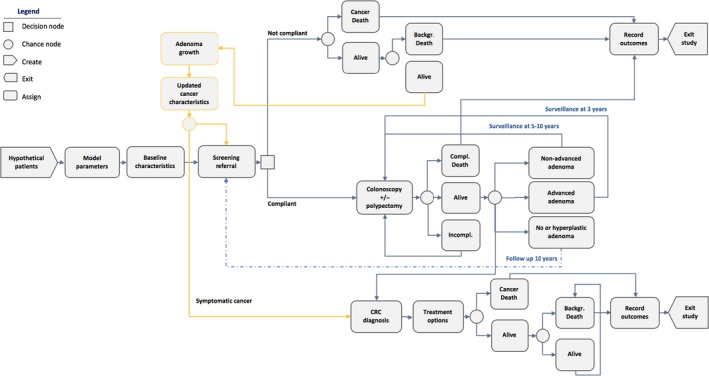
Structure of the screening strategy of colonoscopy every ten years (reference strategy) included in the microsimulation model. The diagram shows the structure of the model for colonoscopy offered every ten years. The yellow lines indicate the time between two subsequent screening referrals in which non‐compliant patients can develop adenomas and/or cancer (COL 10 years). The dotted lines indicate the time to follow‐up. When the time on the dotted lines is not specified in the diagrams, it corresponds to the time interval between two screening referrals of a specific strategy. COL, colonoscopy every ten years

We built a microsimulation model using Arena Version 15.00 (Rockwell Automation Technologies, Inc) that simulates the natural history of CRC, to compare clinical benefits, harms, and burden of testing for alternative strategies for CRC screening (Figure 1 and Figure S1). We created a hypothetical cohort of individuals age 50 years or older, which emulated the distribution of baseline characteristics for subjects in landmark clinical studies (Tables 1).^7^, ^8^ We then created identical cohorts and assigned them to different screening strategies to compare intervention‐related differences in outcomes. For each strategy, we simulated 1000 trials, for a cohort of 10 000 individuals (ie 10 000 000 simulations per strategy). The methods are described in greater detail in the eSupplement. Since changes in CRC incidence, screening performance, and treatment effectiveness over time can impact the assumptions for model input parameters and estimated outcomes, we have presented the specific data sources and study year for all model input parameters in Table 1.

**Table 1:** Model parameters and assumptions

Input parameters	Base case, %	Distribution, range, %	Reference
Natural history of CRC			
Distribution by number of adenomas			Shoenfeld et al 2005 9
1	14.6	±10%	
2	3.8	±10%	
3	1.1	±10%	
4	0.6	±10%	
5	0.5	±10%	
Location of lesions			Atkin et al 2010 7
Proximal	0.34	±10%	
Distal	0.66		
Distribution of lesion type/stage at baseline			
Age and sex specific (see Table S1)			Brenner et al 2015 10
Annual progression rate of lesions			
Age and sex specific (see Table S2)			Brenner et al 2015 10
CRC death rate per year			O’Connell et al 2004 11
Stage I	1.4	±10%	
Stage II	3.5	±10%	
Stage III	8.1	±10%	
Stage IV	18.4	±10%	
Risk of complications			
Risk of any complication during colonoscopy	1.09	1.07‐1.12	Wang et al 2018 22
Risk of serious gastroenterological complications during colonoscopy	0.2	0.19‐0.21	Wang et al 2018 22
One‐time adherence to screening tests			
Flexible Sigmoidoscopy	35%	34.3‐35	Khalid‐de Bakker et al 2011 27
Colonoscopy	38%	25‐55	Singal al. 2017 25
FIT	42.6%	±10%	Akram at al. 2017 26
			Jensen et al 2016 18
HS‐gFOBT	33.4%	±10%	Akram at al. 2017 26
FIT‐DNA	42.6%	±10%	Assumption
CTC	22%	±10%	Khalid‐de Bakker et al 2011 27
SEPT9	85%	±10%	Assumption based on: Adler 2014 15, Liles 2017 16
Adherence to diagnostic colonoscopy^a^	76.2%	74.2‐78.4	Corley et al 2017 17
			Jensen et al 2016 18
Cohort characteristics			Atkin et al 2010 7
Age			
55‐59	50	Not varied	
60‐64	50	Not varied	
Sex			Atkin et al 2010 7
Female	50	Not varied	
Male	50	Not varied	

Abbreviations: CRC, colorectal cancer; CTC computed tomographic colonography; FIT, fecal immunochemical testing; FIT‐DNA, multitarget stool DNA testing; HS‐gFOBT, high‐sensitivity guaiac‐based fecal occult blood test; SEPT9, SEPT9 DNA test.

a Adherence to colonoscopy after a positive non‐invasive test.

### 2.2 Disease progression

The natural history of CRC was a core component of our model. The baseline number of lesions per individual, stage (ie, non‐advanced adenoma, advanced adenoma, preclinical CRC, and clinical CRC) for each lesion and annual progression between stages, stratified by sex and age, were extracted from large observational studies (Tables S1 and S2).^9^, ^10^ We used stage specific CRC death rates from Surveillance, Epidemiology, and End Results (SEER) data to model CRC mortality.^11^

### 2.3 Screening strategies

We evaluated ten different screening strategies (8 screening modalities): (a) no screening (NS); (b) flexible sigmoidoscopy every 5 years (FS); (c) colonoscopy every ten years (COL); (d) annual fecal immunochemical testing (FIT); (e) annual high‐sensitivity guaiac‐based fecal occult blood testing (HS‐gFOBT); (f) multitarget stool DNA testing every 3 years (FIT‐DNA); (g) computed tomographic colonography every 5 years (CTC); and (h) the FDA approved methylated SEPT9 DNA blood test (SEPT9)^12^, ^13^ with 1, 2, and 3 year intervals. All the strategies, other than SEPT9, were included in the recent recommendation statement by the USPSTF.^1^, ^14^ We added the SEPT9 test because evidence suggests it might achieve higher adherence rates compared to endoscopic and fecal‐based tests, and it has been approved by FDA for screening of patients who declined other recommended modalities.^15^, ^16^ Stage‐specific sensitivity and specificity data for all screening tests are shown in Table 2.^13^, ^14^ As done for previous modeling studies,^14^ we assumed that the sensitivity and specificity of all tests remained unchanged over all screening rounds. For sigmoidoscopy, we assumed that the sensitivity was limited to distal lesions. For SEPT9, we collected information on sensitivity and specificity from a prospective study on a sample of 1544 subjects enrolled in the PRESEPT clinical trial, where the clinical performance of SEPT9 was compared to colonoscopy.^13^ We assumed that all screenings started at age 50 years and ended at age 75 years.

**Table 2:** Analytic characteristics of tests and exams used in each screening modality

	Flexible Sigmoidoscopy^a^ (per distal lesion)		Colonoscopy (per lesion)		FIT (per person)^b^		HS‐gFOBT (per person)		FIT‐DNA (per person)		CT Colonography (per person)		SEPT9	
Specificity, %	87	not varied	86	not varied	96.4	not varied	92.5	not varied	89.8	not varied	88	not varied	80.0	77.5‐82.4
Sensitivity, non‐advanced adenomas, %	75	70‐79	75	70‐79	7.6	6.7‐8.6	7.5	not varied	17.2	15.9‐18.6	57	48.9‐71.6	20	15‐23
Sensitivity, advanced adenomas, %	85	80‐92	85	80‐92	23.8	20.8‐27	12.4	10‐26.2	42.4	38.7‐46.2	84	75.6‐92.4	22	18‐24
Sensitivity, cancer, %	95	93.1‐99.5	95	93.1‐99.5	73.8	62.3‐83.3	70	61.5‐79.4	92.3	84‐97	84	75.6‐92.4	68	53‐80

Note

Data on sensitivity and specificity are extracted from Knudsen et al 2016 [ref. 14] for flexible sigmoidoscopy, colonoscopy, FIT, HS‐gFOBT, FIT‐DNA, CT Colonography and from Potter et al 2014 [ref. 13] for SEPT9.

Abbreviations: CT, computed tomographic; FIT, fecal immunochemical testing; FIT‐DNA, multitarget stool DNA testing; HS‐gFOBT, high‐sensitivity guaiac‐based fecal occult blood test; SEPT9, SEPT9 DNA test.

a Sensitivity and specificity are measured only for lesions in the distal colon with % 76‐88 reach.

b FIT characteristics are based on a cutoff for positivity of 100 ng or more of hemoglobin (Hb) per mL of buffer (20 μg Hb/g of feces).

### 2.4 Management after screening

At each pre‐specified screening interval, individuals with negative findings continued to follow the initial screening strategy until age 75 years. Individuals with abnormal findings in CRC screening tests other than colonoscopy were referred for a follow‐up colonoscopy (ie, diagnostic colonoscopy). Abnormal findings were defined as those with true positive and false positive test results based on stage specific sensitivity and specificity for each screening strategy (Table 2). We assumed a 100% adherence to diagnostic colonoscopy when modeling the outcomes under the scenario of 100% adherence to screening strategies. In contrast, we assumed a 76.2% adherence to diagnostic colonoscopy for all screening strategies when modeling outcomes under the reported adherence scenario. Those who did not undergo diagnostic colonoscopy were sent back to their regular screening schedule. The estimate of 76.2% adherence for referrals for diagnostic colonoscopy was derived from published studies on follow‐up colonoscopy after a positive FIT test result.^17^, ^18^

We assumed that any adenoma or CRC lesion detected with colonoscopy was removed during the procedure and patients received the best available post‐intervention treatment. The overall effectiveness of the treatment defined in terms of CRC mortality was already accounted for in the stage‐specific survival rates extracted from SEER data.^11^

The time intervals for subsequent surveillance colonoscopies were modeled following the recommendations of the US Multi‐Society Task Force on Colorectal Cancer^19^ and the National Comprehensive Cancer Network^20^ and varied according to the type of lesions found at the initial diagnostic colonoscopy: one year for patients found with CRC; three years for those with advanced adenoma; five years for those with non‐advanced adenoma; and 10 years for patients with no significant findings. In the model, we assumed 100% adherence to surveillance colonoscopies. All individuals with an abnormal finding by any modality are assumed to be screened exclusively by colonoscopy thereafter. This was done to avoid complexities related to modeling switching between screening modalities.

### 2.5 Time horizon and outcomes

All individuals were followed throughout their lifetime and exited the simulation only for CRC death or background mortality as derived from US life tables published as part of National Vital Statistics Reports.^21^

Primary clinical outcomes included number of CRC cases and CRC deaths. Secondary outcomes included incremental life‐years gained (LYG), number of colonoscopies and tests other than colonoscopy, and number of serious gastrointestinal adverse events (eg, perforation, major bleeding)^22^ related to the use of colonoscopy. The outcomes from each strategy were compared to those of COL, which was the reference strategy.

The efficiency ratio (ER) provides a measure of the benefits from screening (additional LYG) along with the burden of testing (additional number of colonoscopies) required to achieve those gains and is calculated as the incremental number of colonoscopies divided by the incremental LYG (incremental burden to benefit).^14^ We used the ER to compare screening intervals for SEPT9. We did not perform similar analyses for screening intervals of other modalities since they have been already established.^14^

### 2.6 Model validation

We validated the model by comparing: (a) CRC rates and deaths with those reported by the UK Flexible Sigmoidoscopy Screening Trial (UKFSST),^7^ Prostate, Lung, Colorectal, and Ovarian (PLCO) Cancer Screening Trial in the US,^8^ and CISNET models^23^; (b) adenoma dwell time (ie, average time between adenoma incidence and adenoma progression to preclinical cancer) and overall dwell time (ie, average time between adenoma incidence and cancer diagnosis) with those reported by CISNET models^24^; and (c) lifetime risk of developing CRC and CRC deaths with those reported in a large observational study^10^ and CISNET models.^23^ To replicate the UKFSST (conducted between 1994 and 1999), we used the age distribution of trial participants and ran the model over a 10‐year time period to estimate the impact of a one‐time sigmoidoscopy compared to no screening. We ran 1000 simulations for a cohort of 40 000 individuals in each arm to emulate the UKFSST cohort size. Additional details have been provided in the Data S1 section of the eSupplement.

### 2.7 Modeling adherence to screening strategies

We analyzed the outcomes of screening strategies under two scenarios: (a) full (ie, 100%) adherence, where all individuals attend assigned screening strategies; and (b) reported adherence rates.

To model the reported adherence scenario, we derived data on the one‐time adherence rates for each strategy from the published literature (Table 1). One‐time adherence to colonoscopy was estimated at 38%.^25^ The one‐time adherence rates for FIT and HS‐gFOBT were modeled at 42.6% and 33.4%, respectively, based on an observational study of 7898 patients who were offered stool‐based CRC screening.^26^ We assumed that the adherence rate of FIT‐DNA was similar to that of FIT. The adherence rates for FS and CTC were assumed to be 35% and 22%, respectively, and were derived from a systematic review that summarizes attendance rates after initial invitation for different screening tests.^27^ For the SEPT9 blood test, we assumed an 85% one‐time adherence rate in the base case analysis and varied it between 25% and 95% in the sensitivity analyses, as we did for all other strategies. Our assumptions about adherence to SEPT9 were based on results of a randomized trial in the US in which 99.5% of subjects overdue for screening chose to take the blood test,^16^ and a study in Germany in which 83% of patients refusing colonoscopy selected the SEPT9 blood test.^15^

Data from the National Health Interview Survey (NHIS) suggests that up to 62.4% of individuals are currently up to date for CRC screening, primarily via colonoscopy.^28^ This rate was used to calibrate the model to match overall adherence to colonoscopy over 10 years and then the calibrated model was used to simulate adherence patterns for the other screening strategies. This means that, for example, individuals eligible for screening who refuse a colonoscopy in the first year, are offered a colonoscopy in subsequent years such that up to 62.4% of those individuals become up to date within a 10‐year period.

To implement this, we assumed that (a) individuals who are compliant in the first year will have the same one‐time adherence rate in subsequent scheduled screenings (eg adherence would remain 38% for the next round of colonoscopy), and (b) screening was offered every year to non‐compliant individuals with the one‐time adherence rates declining at a constant rate (*r*) each year that they remain non‐compliant (eg 38%*(1 − *r*)*^t^* if colonoscopy delayed by *t* years, where 0 ≤ *t*≤10). Modeling a decline rate was necessary to ensure that the number of simulated individuals up to date with colonoscopy screening would match the observed rate (62.4%)^28^ rather than converging to 100%. We estimated that a 45% decline rate (*r* = 45%) would calibrate the model for colonoscopy. Therefore, we applied the same decline rate to the corresponding one‐time adherence rates of all other screening strategies when individuals were late for screening.

### 2.8 Sensitivity analyses

We conducted a one‐way sensitivity analysis by varying model input parameters between their lowest and highest values to examine the impact on model results. Those values were derived from the literature or were assumed to vary within a 10% range around point estimates when data was absent.

We explored the effect of varying the one‐time adherence rates from 25% to 95% for each screening strategy on cancer incidence and mortality and then compared the results with those of the reference strategy (ie, COL at a 38% one‐time adherence rate).

## 3 RESULTS

### 3.1 Model validation results

The estimated CRC incidence rates over a 10‐year follow‐up period were 149 and 98 per 100 000 person‐year for NS and one‐time sigmoidoscopy, respectively (HR 0.66; 95%CI, 0.58‐0.76) in our model (PREDICT model); vs 149 and 100 per 100 000 person‐year (HR 0.68; 95%CI, 0.60‐0.76) in the UKFSST trial,^7^ and 152 and 119 per 100 000 person‐year (HR 0.79; 95%CI 0.72‐0.85) in the PLCO trial^8^ (Table 3). Estimated CRC mortality and relative hazard ratios were also comparable to the estimates from the UKFSST trial. CRC incidence rate and mortality, as calculated in the three CISNET models, were calibrated using the UKFSST trial, and the reported ranges included the PREDICT estimates (Table 3).

**Table 3:** Results of model validation for 10‐year rates of CRC cases and CRC deaths

	10‐year rate per 100 000 person‐years (95%CI)		HR (95%CI)
	No screening	Flexible sigmoidoscopy	
CRC incidence			
UKFSST Trial^7^	149 (143‐156)	100 (91‐110)	0.68 (0.60‐0.76)
PLCO Trial^8^	152 (144‐160)^a^	119 (112‐127)^a^	0.79 (0.72‐0.85)^a^
CRC‐SPIN^23^	135 (129‐142)	84 (75‐93)	0.62 (0.54‐0.69)
SimCRC^23^	167 (160‐175)	127 (116‐139)	0.74 (0.66‐0.82)
MISCAN^23^, ^b^	183 (175‐191)	160 (147‐173)	0.86 (0.78‐0.94)
PREDICT^c^ (Our model)			
Overall	149 (136‐161)^d^	98 (88‐109)^d^	0.66 (0.58‐0.76)^d^
Distal	99	57	0.57 (0.48‐0.68)
Proximal	50	41	0.84 (0.67‐1.04)
CRC death			
UKFSST Trial^7^	44 (40‐48)	25 (21‐30)	0.56 (0.45‐0.69)
PLCO Trial^8^	39 (35‐43)^a^	29 (25‐32)^a^	0.74 (0.63‐0.87)^a^
CRC‐SPIN^23^	38 (34‐42)	21 (17‐26)	0.57 (0.44‐0.71)
SimCRC^23^	52 (48‐57)	33 (28‐39)	0.63 (0.52‐0.76)
MISCAN^23^, ^b^	37 (34‐41)	25 (21‐30)	0.68 (0.53‐0.83)
PREDICT^c^			
Overall	41 (35‐48)^d^	26 (20‐31)^d^	0.63 (0.48‐0.80)^d^
Distal	27	15	0.54 (0.38‐0.74)
Proximal	14	11	0.83 (0.53‐1.24)

a The benefits of the PLCO trial are partially contaminated using colonoscopy in both arms.

b The MISCAN model has been updated and the results of the re‐calibrated model can be found in Rutter et al 2016 [ref. 23].

c Predictive modeling, Evidence integration, and Decision analysis In Clinical Therapeutics (PREDICT) group, Division of Pharmacoepidemiology and Pharmacoeconomics, Brigham and Women's Hospital, Harvard Medical School.

d 95% credible intervals reflect variation of mean values across 1000 simulated trials, each including a cohort of 40 000 screened individuals. The cohort size of 40 000 individuals was chosen to emulate the size of the screened group in the UKFSST trial.

The estimated adenoma dwell time and overall dwell time were 20.5 years and 26.2 years, respectively (Table S3A), consistent with those reported by two CISNET models (SimCRC and CRC‐SPIN).^24^ The lifetime risks of CRC incidence and CRC mortality predicted by our model under NS (64 cases and 30 deaths per 1000 individuals) were in line with the rates reported by the CISNET models (67‐72 cases and 27 to 28 deaths per 1000 individuals) (Table S3A). Additional validation data are presented in Table S3B and C (10‐year CRC incidence, detection rate of adenomas, referrals to colonoscopy and CRC detection rate at screening). Age‐specific CRC incidence and mortality are shown in Figure S2.

### 3.2 Results assuming full adherence

**Figure 2:**
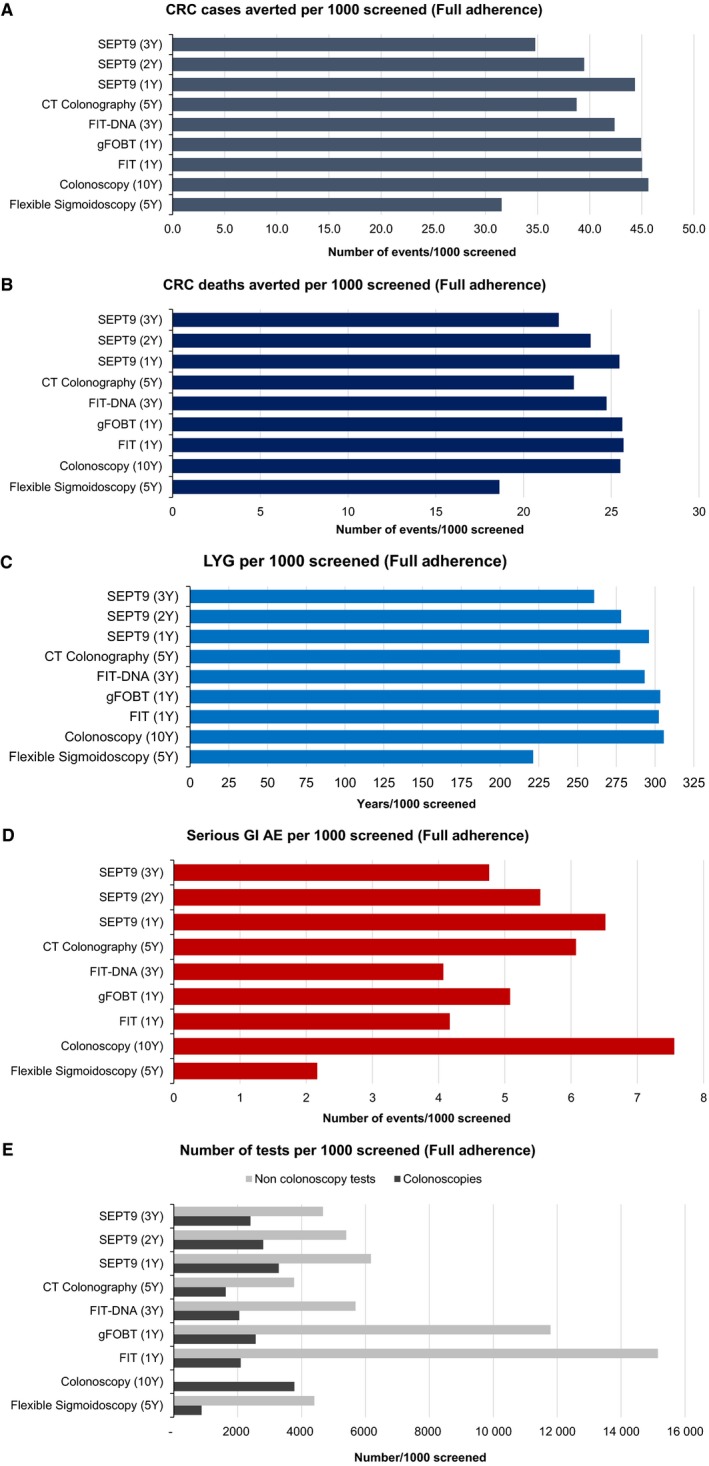
The graphs show the numbers of colorectal cancer cases and colorectal cancer deaths averted, life years gained, and gastrointestinal adverse events due to endoscopic procedures across all the available screening strategies (compared with NS), assuming full (i.e., 100%) adherence. CRC, colorectal cancer; LYG, life‐years gained; GI, gastrointestinal; AE, adverse events; FIT (1Y), fecal immunochemical testing every year; gFOBT (1Y), high‐sensitivity guaiac‐based fecal occult blood test every year; FIT‐DNA (3Y), multitarget stool DNA testing every three years; CT Colonography (5Y), computed tomographic colonography every five years; SEPT9 (1Y), SEPT9 DNA test every year; SEPT9 (2Y), SEPT9 DNA every two years; SEPT (3Y), SEPT9 DNA every three years

Number of CRC cases and CRC deaths averted, LYG, and number of gastrointestinal adverse events due to endoscopic procedures across all the screening strategies (compared with NS), assuming 100% adherence, are presented in Figure 2. FIT‐DNA every three years, FIT, HS‐gFOBT, and SEPT9 annually averted 42 to 45 cases and 25 to 26 deaths per 1000 individuals screened; COL averted 46 CRC cases and 26 CRC deaths and CTC averted 39 CRC cases and 23 CRC deaths per 1000 individuals screened; and FS averted 32 CRC cases and 19 CRC deaths per 1000 individuals screened. Estimated LYG were similar across FIT‐DNA, FIT, HS‐gFOBT, SEPT9, CTC, and COL strategies.

The total number of colonoscopies was highest in the COL strategy (3777), followed by SEPT9 every year (3286), HS‐gFOBT (2559), FIT‐DNA (2048), FIT (2095), CTC (1623) and FS (871) (Figure 2 and Table S4). The number of adverse events (perforation or major bleeding) was proportional to the number of colonoscopies and was highest in the COL strategy (7.6 per 1000 individuals screened), followed by SEPT9 every year (6.5), CTC (6.1), HS‐gFOBT (5.1), FIT‐DNA (4.1), FIT (4.2), and FS (2.2).

ERs of annual vs two‐ and three‐year intervals for SEPT9 were 27 and 25 additional colonoscopies per LYG, respectively. Because both the USPSTF and the American Cancer Society consider an ER equal to 39 or less as an acceptable number of incremental colonoscopies per LYG,^14^, ^29^ the annual SEPT9 resulted as the optimal SEPT9 strategy.

### 3.3 Results assuming reported adherence rates

**Figure 3:**
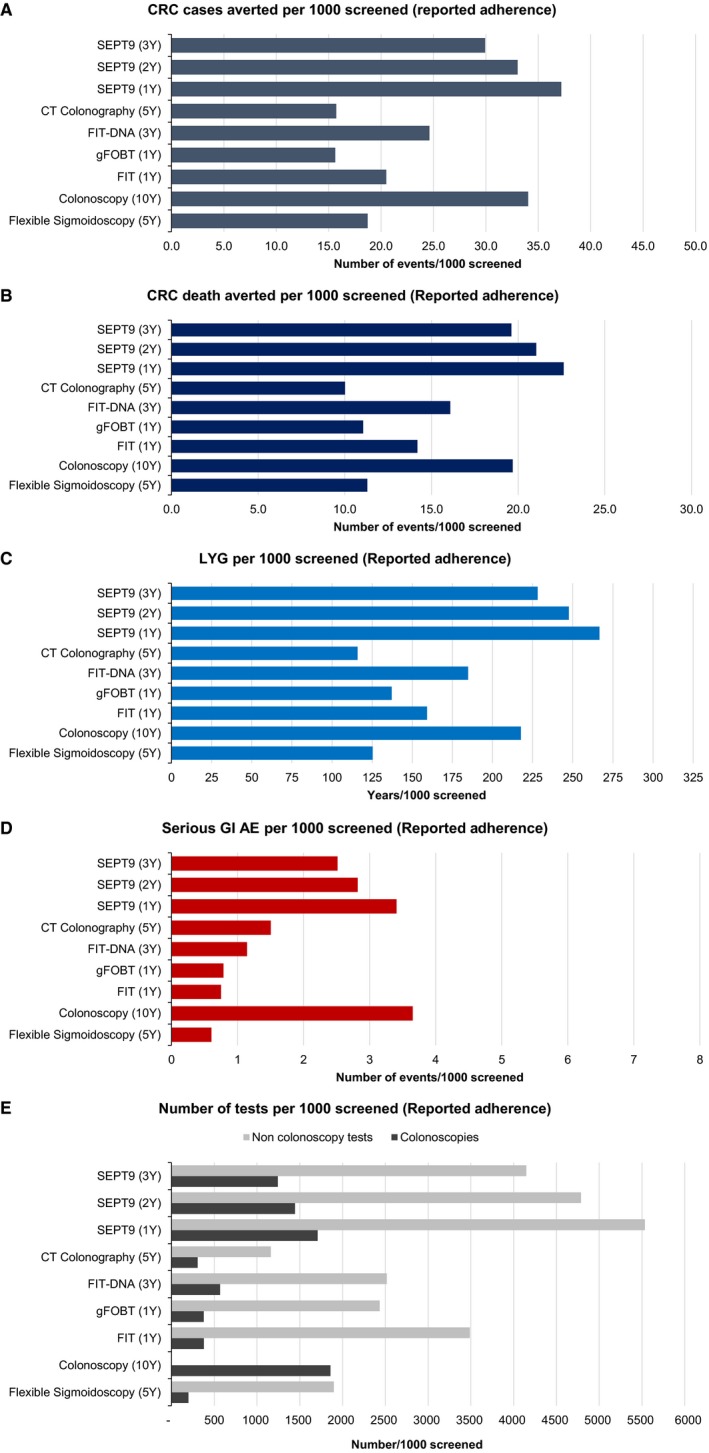
The graphs show the numbers of colorectal cancer cases and colorectal cancer deaths averted, life years gained, and gastrointestinal adverse events due to endoscopic procedures across all the available screening strategies (compared with NS), assuming full (i.e., 100%) adherence. CRC, colorectal cancer; LYG, life‐years gained; GI, gastro intestinal; AE, adverse events; FIT (1Y), fecal immunochemical testing every year; gFOBT (1Y), high‐sensitivity guaiac‐based fecal occult blood test every year; FIT‐DNA (3Y), multitarget stool DNA testing every three years; CT Colonography (5Y), computed tomographic colonography every five years; SEPT9 (1Y), SEPT9 DNA test every year; SEPT9 (2Y), SEPT9 DNA every two years; SEPT (3Y), SEPT9 DNA every three years

When we applied published adherence rates, SEPT9 every year averted 37 CRC cases and 23 CRC deaths per 1000 individuals screened; COL averted 34 CRC cases and 20 CRC deaths per 1000 individuals screened; FS, FIT‐DNA, FIT, CTC and HS‐gFOBT averted approximately 16 to 25 CRC cases and 11 to 16 CRC deaths per 1000 individuals screened (Figure 3). LYG reflected the effectiveness of each strategy in reducing CRC cases and deaths.

As expected, in this scenario the number of colonoscopies for each strategy was lower compared with the scenario of full adherence (Figure 3 and Table S5). The total number of colonoscopies was highest in the COL strategy (1897), followed by SEPT9 every year (1708), FIT‐DNA (568), FIT (379), HS‐gFOBT (377), CTC (305), and FS (199) (Figure 3 and Table S5). The number of adverse events (perforation or major bleeding) was highest in the COL strategy (3.7 per 1000 individuals screened), followed by SEPT9 every year (3.4), CTC (1.5), FIT‐DNA (1.1), HS‐gFOBT (0.8), and FIT (0.8), and FS (0.6),

The total number of non‐colonoscopy and colonoscopy tests performed over time for each screening strategy under both reported and full adherence scenarios is presented in Figure S3.

### 3.4 Sensitivity analyses

Results of the sensitivity analyses from varying one‐time adherence rates from 25% to 95% for all modalities (compared to COL, as reference strategy) are shown in Figure S4. The findings suggested that at adherence rates of 65%‐70% or higher for FIT, HS‐gFOBT, FIT DNA, CTC, and annual SEPT9, CRC cases and CRC deaths averted would equal or exceed those of COL at its reported one‐time adherence rate (38%).

The sensitivity analysis of the one‐time adherence rate for COL (25% and 95%) showed that the effect on cancer incidence and mortality was relatively modest as compared to the one‐time adherence rate used in the base case analysis (38%) (Figure S4). This seems plausible since we have assumed that colonoscopy was offered every year to non‐compliant individuals, who had a longer time window to comply with scheduled colonoscopy compared to the other screening tests offered at shorter intervals.

## 4 DISCUSSION

We developed a microsimulation model to compare the clinical benefits, harms and burden of testing across alternative screening strategies, assuming full adherence (ie, 100%) or reported adherence rates in the scientific literature. Our results indicated that adherence has a substantial impact on clinical outcomes and should influence the selection of optimal screening strategies. In the ideal condition of full adherence, screening with stool‐based tests (ie, FIT‐DNA, FIT, HS‐gFOBT), CT colonography, or annual SEPT9 would produce comparable benefits to screening with colonoscopy every ten years. However, when we modeled reported adherence rates, the results changed substantially: colonoscopy appears superior in reducing cancer incidence and mortality over all other USPSTF recommended CRC screening methods while annual SEPT9 is predicted to be an effective non‐invasive option for patients unwilling or unable to use the other modalities. The findings suggested that adherence rates higher than 65%‐70% would be required for any stool or blood‐based screening modality to achieve the benefits of colonoscopy.

As mentioned above, at 100% adherence, the stool‐based tests and SEPT9 showed a clinical performance similar to that of colonoscopy. Although these tests have a substantial lower one‐time sensitivity than colonoscopy, the frequent testing and shorter test interval increases the overall probability of cancer detection over time. A subset of interval cancers has been reported to occur in the ten‐year interval between screening colonoscopies.^29^ In addition, the results of the stool‐ and blood‐based tests might be slightly overestimated because we did not model a decline in sensitivity over time. For example, some evidence shows that FIT sensitivity for CRC—as well as for adenoma—is higher at the first screening round, decreases at the second, and remains approximately stable in the subsequent rounds.^17^, ^30^ However, since FIT screening is shown to be more sensitive for later stage CRCs,^31^ this decline likely reflects a shift in the detection of a higher number of prevalent, later stage cancer cases in the first round of screening (detection of prevalent cancer) as compared to a lower number of such cases in subsequent rounds (detection of incident cancer).

In line with the current literature,^2^ our findings show that the impact of adherence is substantial when compared to the impact of analytic performance on screening outcomes. Knudsen and colleagues^31^ conducted a simulation study to assess the benefits of rescreening with alternative strategies individuals who had a negative result on the screening colonoscopy vs continuing screening colonoscopy every ten years. They found that benefits of alternative rescreening strategies were sensitive to assumptions about adherence rates. Ladabaum and Mannalithara^32^ developed a simulation model to compare the benefits of FIT vs a stool DNA test that has higher sensitivity but lower specificity than FIT. Their results also suggested that the pattern of adherence had a large impact on the relative effectiveness of those tests. Similarly, the favorable outcomes demonstrated in our study for SEPT9, compared with other non‐colonoscopy screening tests, were due to the higher reported compliance. A prospective primary care study conducted in Germany, reported that out of 172 subjects who were referred to screening, only 63 (37%) were compliant to screening with colonoscopy.^15^ Within the remaining 109 patients who refused colonoscopy, 90 (83%) decided to take the SEPT9 DNA blood test. This suggests that offering a blood‐based test has the potential to improve overall patient adherence. Patient preference for the blood test has also been demonstrated in a randomized trial in the US which compared the adherence of the blood‐based test with FIT (99% vs 88%).^16^ However, additional data on compliance are needed to establish the actual adherence rates in a real‐world setting, and across alternative screening options. Blood tests might be more adaptable to incorporation into scheduled annual physical exams where blood draws are common. Moreover, the lower specificity of SEPT9 did not lead to a greater number of harmful events when compared to colonoscopy.

Few studies have attempted to model the impact of adherence on screening outcomes, assessing a different research question (eg, analysis of rescreening modalities)^31^ or providing direct comparisons of two strategies.^32^, ^33^ Models comparing several screening strategies have assumed full patient adherence^1^, ^14^ or have only peripherally examined the impact of lower adherence rates across all modalities.^34^ Our aim was to fill this gap by accounting for reported adherence rates for each screening strategy and providing more realistic estimates on the relative effectiveness. Because of the uncertainty of the adherence estimation, we performed several sensitivity analyses testing different scenarios. The impact of adherence on outcomes for all screening methods clearly demonstrates the critical importance of adherence in developing screening strategies.

Our model closely replicates the outcomes observed in the no screening and intervention arms of a large randomized controlled trial conducted in the UK (UKFSST) and provides comparable results to the trial conducted in the US (PLCO).^7^, ^8^ This was important to ensure that our model has been correctly specified and can accurately simulate the natural history of CRC and the impact of screening interventions on clinical outcomes. Our model used the data on transition rates from a recently published study on a large data registry.^10^, ^35^, ^36^ However, we acknowledge the challenges involved in replicating trial results because not all information required for accurate modeling is available. In the PLCO trial, a large proportion of individuals under usual care received flexible sigmoidoscopy or colonoscopy out of study protocol. Because of this contamination, the estimated relative risk reduction in the PLCO trial was smaller compared to the UKFSST trial. Also, only 71.2% and 86.6% of individuals who were randomized to sigmoidoscopy in the PLCO and UKFSST trials, respectively, underwent screening. Because in the simulation we assumed that all individuals in the FS group received one flexible sigmoidoscopy, we compared the results of our validation with UKFSST trial results that are separately reported for the subgroup who underwent screening in the intervention arm per protocol (n = 40 621). For these reasons, our validation results closely resemble those reported as part of the UKFSST trial (see Table 3).

Our model has several limitations. First, the observational study that we used for modeling disease progression only reported transition rates between four stages: non‐advanced adenomas, advanced adenomas, pre‐clinical CRC, and clinical CRC.^10^ Therefore, the granularity of our model for adenoma size and characteristics is limited. However, the advantage of using observed transition rates outweighs the limited granularity and the need for estimating the rates using calibration approaches. In addition, this study is conducted in a German population that might differ from the US population. However, CRC burden appears to be similar in Germany and the US.^37^ Second, although we acknowledge that the serrated pathway accounts for approximately 20 to 30% of colorectal cancer,^38^ we did not consider modeling it due to lack of data and associated differences in disease progression. Because these lesions may represent a distinct cancer pathway with different transition times, exclusion could affect the predicted cancer rate. However, since this group is included in the advanced adenoma classification, the impact is expected to be limited. Third, in the absence of data on the programmatic performance of screening modalities, we assumed that the sensitivity and specificity of all tests remained constant. This limitation can affect our relative findings, potentially overestimating sensitivity, but also overestimating the false referral rate. It would have a smaller impact on tests with longer intervals, such as colonoscopy and CTC, compared to tests with shorter intervals. Fourth, in the evaluation of reported adherence rates, we assumed that individuals remained adherent to their initial screening modality over time. In clinical practice, patient adherence may vary from year to year, and these gaps in continuous screening are more likely for modalities with shorter time intervals. Thus, the clinical benefits gained using strategies such as stool‐based tests and the SEPT9 blood test might be slightly overestimated in our model. Fifth, cost‐effectiveness is an important aspect that should be considered when comparing different screening strategies. In a three year budget impact simulation, it was reported that FIT‐ DNA and SEPT9 resulted in similar costs while FIT was less expensive.^39^ In future studies our model will be extended to account for the cost and cost‐effectiveness of different screening strategies. Performing a probabilistic sensitivity analysis will also help demonstrate variation of estimated outcomes due to uncertainty in model input parameters. Finally, data on studies that have reported on adherence rates are limited and can vary across different populations, healthcare systems, and geographies.^26^, ^27^, ^28^, ^31^, ^40^, ^41^, ^42^ Given these caveats, we performed several sensitivity analyses and provided information on the clinical outcomes for each screening modality at different adherence rates.

In summary, this study highlights the key impact that adherence has on the effectiveness of alternative screening modalities for colorectal cancer. We found that higher expected adherence rates can lead to meaningful benefits when compared to strategies with better one‐time sensitivity and/or specificity, but lower adherence rates. This is relevant for optimizing screening strategies when considering other factors in addition to test performance, such as access to screening and patient behavior. We expect that more evidence will be accumulated in support of our findings.

## CONFLICT OF INTEREST

Najafzadeh was a consultant to Epigenomics during the conduct of this study. The other authors do not have any conflicts of interests.

## Data Availability

All data used to build the model are referenced throughout the manuscript and are made available upon reasonable request.
