# Supplementary material for: Quantifying the impact of adherence to screening strategies on colorectal cancer incidence and mortality
Source: Cancer Med. 2019 Nov 28;9(2):824–36. doi: 10.1002/cam4.2735 (PMC6970061; doi:10.1002/cam4.2735)
Supplement: Supplementary file 1 [file CAM4-9-824-s001.docx]

**eSupplement**

**eMethods**

**eTable 1.** Baseline distribution of lesion types by age and sex.

**eTable 2.** Annual transition rates between different lesion stages by age and sex.

**eTable 3.** Model validation results.

**eTable 4.** Outcomes of screening strategies assuming 100% adherence rates.

**eTable 5.** Outcomes of screening strategies assuming reported adherence rates.

**eFigure 1.** Model structure for different screening strategies.

1. FIT, HS-gFOBT, FIT-DNA, and SEPT9 screening strategies
2. CT colonography screening strategy
3. Flexible sigmoidoscopy screening strategy
4. Modeling natural history of disease in the microsimulation

**eFigure 2.** Model validation results: estimated age-specific CRC cases and CRC deaths.

**eFigure 3**. The total number of non-colonoscopy and colonoscopy tests performed over time under the full adherence and reported adherence scenarios for each screening strategy.

**eFigure 4.** One-way sensitivity analyses: colorectal cancer cases and deaths averted across the screening strategies for different adherence rates as compared to colonoscopy every ten years with reported adherence rates.

**eSupplement**

In this Supplement, we provide more information regarding the model structure (eFigures 1 A to C), modeling the natural history of CRC (eFigure 1D), assumptions, data sources, and additional analyses that did not appear in the main version of the manuscript because of space limitations.

**eMethods**

***Disease Progression***

Modeling and validating the natural history of CRC was a core component of our microsimulation model. The baseline lesion stage, defined as a specific status of a colorectal cancer lesion (i.e., non-advanced adenoma, advanced adenoma, or pre-clinical CRC), was assigned according to the distributions of age and sex extracted from a large observational study [^1^](#_ENREF_1). This study reported age and sex distributions for each CRC stage based on a sample of more than 4.4 million colonoscopies, captured in the national colonoscopy screening registry from 2003 through 2012 in Germany (eTable 1). It also estimated annual age and sex specific progression rates between different disease stages (i.e., non-advanced adenoma, advanced adenoma, preclinical CRC, and clinical CRC). We used those transition rates to model disease progression over time as a function of individuals’ age, sex, and lesion stage (eTable 2). The baseline estimates of age and sex specific percentages of individuals with any adenoma or preclinical cancer were derived from a large observational study.[^1^](#_ENREF_1) The number of lesions per individual, conditional on having any adenoma or cancer, in our hypothetical cohort emulated the actual distribution in a large colonoscopy screening study performed in the US[^2^](#_ENREF_2). In this study, among 1,463 participants who underwent colonoscopy, 300 (20.6%) had at least one neoplastic lesion. We assumed that at the baseline 14.6%, 3.8%, 1.1%, 0.6%, and 0.5% of individuals with lesions in our model had 1, 2, 3, 4, and 5 lesions, respectively. We assumed that approximately 67% of lesions were located in the distal colon[^3^](#_ENREF_3). In line with the definition used in The Prostate, Lung, Colorectal, and Ovarian (PLCO) Cancer Screening trial, we defined distal location as the rectum through the splenic flexure, and proximal as the transverse colon through the cecum[^4^](#_ENREF_4). Each lesion was characterized at the baseline by stage (non-advanced adenoma, advanced adenoma, pre-clinical CRC, or clinical CRC) and location (distal, proximal) and its progression over time was simulated independently of other lesions that might be present in the same individual. We defined a patient’s disease stage based on his/her most advanced lesion at any given time point. Progression of clinical CRC stage I to more advanced stages (II to IV) was modeled assuming a constant annual transition rate. In the absence of reliable data sources, this transition rate was estimated through calibration. We used stage specific CRC mortality rates observed in the nationally representative Surveillance, Epidemiology, and End Results (SEER) data to model death due to CRC[^5^](#_ENREF_5).

Our assumption of a constant progression rate from stage I to stage IV clinical CRC might affect CRC death estimates. However, this was a necessary assumption considering the lack of observational data on the progression of clinical cancer. Importantly, the assumption of constant progression does not influence cancer incidence.

***Model Validation***

We validated the model by comparing our findings, in terms of CRC rates and CRC deaths, for no screening and flexible sigmoidoscopy (FS) strategies with a landmark randomized trial: the UK Flexible Sigmoidoscopy Screening Trial (UKFSST)[^3^](#_ENREF_3). Although UKFSST was chosen as our target trial for replication, we also compared our results with PLCO in the US[^4^](#_ENREF_4) and with those reported in the three CISNET models [^6^](#_ENREF_6).

***Model assumptions for validation***

We used the age and sex distribution of participants in the UKFSST trial to create a cohort similar to trial participants (see cohort characteristics in Table 1). In line with the UKFSST trial, we assumed that individuals in the screening group received only one flexible sigmoidoscopy within the first year of simulation. Individuals with adenomas larger than 10mm, those with 3 or more adenomas, and those with pre-clinical cancer identified during screening were referred to diagnostic colonoscopy. We assumed that 76.2% of those received a diagnostic colonoscopy within the same year.

In the PLCO trial, a large proportion of individuals under usual care received flexible sigmoidoscopy or colonoscopy out of study protocol. Because of this contamination, the estimated relative risk reduction in the PLCO trial was smaller compared to the UKFSST trial. Also, only 71.2% and 86.6% of individuals who were randomized to sigmoidoscopy in the PLCO and UKFSST trials, respectively, underwent screening. Given that in our simulation we assumed that all individuals in the FS group received one flexible sigmoidoscopy, we compared the results of our validation with UKFSST trial results that are separately reported for the subgroup who underwent screening in the intervention arm per protocol (n=40,621). For these reasons, our validation results closely resemble those reported as part of the UKFSST trial (see Table 3).

We used specificity and stage and location specific sensitivities consistent with those used in the CISNET models (Table 2). We assumed that flexible sigmoidoscopy can only visualize the distal colon and with 76-88% reach. We defined distal and proximal locations according to the PLCO trial.

We assumed subsequent surveillance colonoscopies were offered after one year for patients found with CRC; three years for those with advanced adenoma; and five years for those with non-advanced adenoma and followed with 100% adherence.

We simulated 1000 trials, each trial included 40,000 individuals in each study arm and followed them up for 10 years.

***Model calibration***

Because we used the transition rates between different adenoma stages directly from recently published data from a large data registry,[^1^](#_ENREF_1)^,^[^7^](#_ENREF_7)^,^[^8^](#_ENREF_8) there was minimal need to do a model calibration. The calibration only involved dividing all the age and sex specific transition rates by a constant (1.38). In the absence of reliable data sources, progression of CRC stage I to more advanced stages (II to IV) was modeled assuming a constant annual transition rate that was estimated through calibration (10% per year for all stages I to IV).

We compared our estimated dwell times (defined as the average time between adenoma incidence and adenoma progression to preclinical cancer - adenoma dwell time, or the average time between adenoma incidence and cancer diagnosis - overall dwell time) with those of the CISNET models [^9^](#_ENREF_9). Finally, we compared life-time risk of developing CRC and CRC deaths with the rates reported in a large observational study[^1^](#_ENREF_1) and in the CISNET models[^10^](#_ENREF_10).

We assumed that all pre-clinical CRCs at baseline were cancers at stage I.

***Cohort characteristics***

Similar to the three CISNET models, PREDICT models the natural history of colorectal cancer based on the adenoma-carcinoma sequence depicted in eFigure 1D (Supplement). In PREDICT, simulated persons start with or without lesions (i.e. non-advanced adenoma, advanced adenoma, preclinical CRC) at age 50 and may progressively transition to more advanced states, including clinically detected CRC or CRC death. Individuals may also die because of age and sex-specific background mortality. Because PREDICT has been designed based on transition rates estimated from a large observational study[^1^](#_ENREF_1), we chose 50 as the starting age for modeling the natural history of disease. The three CISNET models do not allow detectable adenomas in individuals younger than 20 years of age and begin to model progression of disease at that point through the same disease states. In all four models, the adenoma risk varies stochastically across individuals and multiple adenomas may be generated within an individual. None of the model allow regression of adenomas, nor simulate the serrated polyp pathway for CRC.

The models are calibrated so that model predictions closely match observational data. The CISNET models are calibrated by adjusting various parameters including but not limited to the risk of a lesion occurring, its size, growth rate, number and distribution, transition rates, according to sex, age, location along the large intestine and other factors to best fit the age-specific CRC incidence and related mortality rates reported from observational studies and other sources such as SEER data. The CISNET models then analyze a cohort of individuals starting at age 40 to measure the predicted incidence and mortality rates of CRC until death. For the USPSTF analysis, they model various screening intervention strategies with the current recommendation starting at age 50 years and ending at 75 years. PREDICT takes a different approach, modeling individuals starting at age 50 years, deriving the age and sex specific distribution of lesions and cancer and age and sex specific transition rates from a large observational study of over 4.4 million colonoscopies. The baseline for the natural history of disease starts at age 50 and progresses in those with disease until death from CRC or other causes. Screening intervention is modeled starting at age 50 years and ending at 75 years as well.

Although PREDICT starts the simulation of natural history of CRC at age 50 years (in contrast to age 20 for CISNET models), this difference only impacts how the models generate a baseline of individuals for entry into the model. The key is for the prevalence of lesions (adenomas) and pre-clinical cancer at the start of simulation and the incidence of CRC and CRC death during the period of intervention (age 50-75 years) to be comparable to observational trial data. In support of this, the dwell times, CRC incidence, mortality rates, and simulated adenoma prevalence are comparable between PREDICT, the CISNET models and those reported from two large observational studies (UKFSST and PLCO). Therefore, although the models are built differently and have a different starting age for simulating the natural history of CRC, the predicted outcomes are similar for all models.

**eTable 1.** Model input parameters: baseline distribution of lesion types by age and sex.

|  | **Age** | **No neoplasm (%)** | **Non-advanced adenoma (%)** | **Advanced adenoma (%)** | **CRC (%)** |
| --- | --- | --- | --- | --- | --- |
| **Men** |  |  |  |  |  |
|  | 55–59 | 75.6 | 17.2 | 6.7 | 0.6 |
|  | 60–64 | 72.1 | 18.4 | 8.4 | 1.0 |
|  | 65–69 | 69.8 | 19.5 | 9.4 | 1.4 |
|  | 70–74 | 67.7 | 20.3 | 10.1 | 2.0 |
|  | 75–79 | 67.4 | 19.5 | 10.6 | 2.6 |
|  | 80–84 | 68.1 | 18.1 | 10.4 | 3.5 |
| **Women** |  |  |  |  |  |
|  | 55–59 | 85.7 | 10.4 | 3.6 | 0.3 |
|  | 60–64 | 83.2 | 11.6 | 4.7 | 0.5 |
|  | 65–69 | 81.1 | 12.8 | 5.4 | 0.7 |
|  | 70–74 | 78.7 | 14.0 | 6.2 | 1.1 |
|  | 75–79 | 77.8 | 13.8 | 6.8 | 1.6 |
|  | 80–84 | 76.6 | 13.1 | 7.5 | 2.7 |

Source: Brenner et al (2015), Clinical Gastroenterology and Hepatology.

**eTable 2.** Model input parameters: annual transition rates between different lesion stages by age and sex**.**

|  | **Non-advanced to advanced adenoma (%)** | **Advanced adenoma to pre-clinical cancer (%)** | **Preclinical cancer to clinical cancer (%)** |
| --- | --- | --- | --- |
| **Men** |  |  |  |
| 55–59 | 4.2 (3.8–4.6) | 2.6 (2.4–2.9) | 18.1 (16.7–19.5) |
| 60–64 | 4 (3.6–4.4) | 3.1 (2.8–3.3) | 19.2 (18.1–20.3) |
| 65–69 | 4 (3.6–4.3) | 3.8 (3.5–4.1) | 21.3 (20.3–22.4) |
| 70–74 | 4.1 (3.6–4.6) | 5.1 (4.8–5.5) | 20.6 (19.5–21.7) |
| 75–79 | 3.7 (2.9–4.6) | 5.2 (4.6–5.8) | 20.1 (18.9–21.4) |
| 80–84 | 3.7 (2.9–4.6) | 5.2 (4.6–5.8) | 18.2 (16.7–19.9) |
| ≥85 | 3.7 (2.9–4.6) | 5.2 (4.6–5.8) | 18.2 (16.7–19.9) |
| **Women** |  |  |  |
| 55–59 | 4 (3.6–4.5) | 2.5 (2.2–2.7) | 21.3 (19.5–23.4) |
| 60–64 | 3.6 (3.2–4.1) | 2.7 (2.4–3.0) | 22.5 (20.9–24.2) |
| 65–69 | 3.7 (3.2–4.1) | 3.8 (3.5–4.1) | 21.9 (20.6–23.3) |
| 70–74 | 4.7 (4.1–5.3) | 5 (4.5–5.4) | 20.8 (19.4–22.2) |
| 75–79 | 3.7 (2.8–4.7) | 5.6 (4.9–6.3) | 19.2 (17.9–20.7) |
| 80–84 | 3.7 (2.8–4.7) | 5.6 (4.9–6.3) | 17.3 (16.0–18.8) |
| ≥85 | 3.7 (2.8–4.7) | 5.6 (4.9–6.3) | 17.3 (16.0–18.8) |

Source: Brenner et al (2015), Clinical Gastroenterology and Hepatology.

**eTable 3. Model validation results**

**A.** Model validation results: estimated dwell time and life-time risk of CRC cases and CRC deaths

|  | **SimCRC** | **MISCAN** | **CRC-SPIN** | **PREDICT** ^&^ **(Our model)** |
| --- | --- | --- | --- | --- |
| **Estimated dwell times*** | | | | |
| Non-advanced Adenoma to Pre- Clinical Cancer | 21.2 (12 – 29) | 7.6 (2- 11)** | 24.2 (16 – 31) | 20.5 |
| Non-advanced Adenoma to Clinical Cancer | 25.2 (15 – 33) | 10.6 (5 – 14) | 25.8 (17 – 33) | 26.2 |
| **Estimated life time risk of events (per 1000)** | | | | |
| CRC | 70 | 67 | 72 | 64*** |
| CRC death | 28 | 28 | 27 | 30 |

^&^Predictive modeling, Evidence integration, and Decision analysis In Clinical Therapeutics (PREDICT) group, Division of Pharmacoepidemiology and Pharmacoeconomics, Brigham and Women’s Hospital, Harvard Medical School

*Source: Kuntz et al (2011), Medical Decision Making

Lifetime risk of CRC cases and CRC deaths for CISNET models are for a cohort of 40 years old. Lifetime risks for our model are for a cohort of 50 years old.

** The MISCAN model has been updated and the results of re-calibrated model can be found in Rutter et al (2016)

***Lifetime risk of CRC has been estimated to be 7.5% for men and 6.1% for women in Germany. Source: Brenner et al (2015), Clinical Gastroenterology

**B.** Model validation results: CRC cases and CRC deaths at 10 years predicted by different models as compared to UKFSST trial.

|  | **10-year CRC incidence rate per 100,000 person-years (95%CI), No Screening** | |  |
| --- | --- | --- | --- |
|  | **CRC rate per 100,000** | **95% Interval** | **Interval width** |
|  |  |  |  |
| **Overall** |  |  |  |
| UKFSST Trial[^4^](#_ENREF_4) | 149 | (143-156) | 13 |
| PLCO Trial[^4^](#_ENREF_4)^#^ | 152 | (144-160) | 16 |
| CRC-SPIN[^6^](#_ENREF_6) | 135 | (129-142) | 13 |
| SimCRC^[6](#_ENREF_6)^ | 167 | (160-175) | 15 |
| MISCAN[^6^](#_ENREF_6) | 183 | (175-191) | 16 |
| PREDICT | 149 | (136 -161) | 25 |
| **Distal** |  |  |  |
| UKFSST Trial[^4^](#_ENREF_4) | 98 | (92- 103) | 11 |
| PLCO Trial[^4^](#_ENREF_4)^#^ | 79 | (73- 85) | 12 |
| CRC-SPIN[^6^](#_ENREF_6) | 64 | (59- 69) | 10 |
| SimCRC^[6](#_ENREF_6)^ | 116 | (109- 122) | 13 |
| MISCAN[^6^](#_ENREF_6) | 98 | (92- 103) | 11 |
| PREDICT | 99 | (90- 109) | 19 |
| **Proximal** |  |  |  |
| UKFSST Trial[^4^](#_ENREF_4) | 51 | (48- 56) | 8 |
| PLCO Trial[^4^](#_ENREF_4)^#^ | 70 | (65- 76) | 11 |
| CRC-SPIN[^6^](#_ENREF_6) | 71 | (66- 77) | 11 |
| SimCRC^[6](#_ENREF_6)^ | 51 | (47- 56) | 9 |
| MISCAN[^6^](#_ENREF_6) | 85 | (80- 90) | 10 |
| PREDICT | 50 | (43- 57) | 14 |

^&&^The MISCAN model has been updated and the results of re-calibrated model can be found in Rutter et al (2016) online eSupplement

^#^The benefits of the PLCO trial are partially contaminated by the use of colonoscopy in both arms.

**C.** Model validation results: outcomes at screening for PREDICT compared to UKFSST trial and CISNET models

|  | **%** | **95% Interval** |
| --- | --- | --- |
| **Adenomas detected at flexible sigmoidoscopy*** |  |  |
| UKFSST Trial | 12.1 | (11.8 - 12.4) |
| CRC-SPIN | 9.4 | (9.2 - 9.7) |
| SimCRC | 8.8 | (8.5 - 9) |
| MISCAN | 22.7 | (22.4 - 23.1) |
| PREDICT | 9.3 |  |
| **Referred to colonoscopy** |  |  |
| UKFSST Trial | 5.2 | (4.3 - 6.2) |
| CRC-SPIN | 3.6 | (3.4 - 3.8) |
| SimCRC | 4 | (3.8 - 4.2) |
| MISCAN | 7.2 | (7.0 - 7.5) |
| PREDICT | 4.5 |  |
| **Adenomas detected at colonoscopy** |  |  |
| UKFSST Trial | 18.8 | (17.1 - 20.5) |
| CRC-SPIN | 39.1 | (36.7 - 41.7) |
| SimCRC | 35.6 | (33.4 - 37.9) |
| MISCAN | 45.6 | (43.8 - 47.3) |
| PREDICT | 28.4 |  |
| **CRC detected at screening** |  |  |
| UKFSST Trial | 0.34 | (0.29 - 0.40) |
| CRC-SPIN | 0.11 | (0.08 - 0.15) |
| SimCRC | 0.44 | (0.38 - 0.51) |
| MISCAN | 0.43 | (0.37 - 0.49) |
| PREDICT | 0.48 |  |

*Rutter et al (2016) Medical Decision Making.

**eTable 4.** Outcomes of screening strategies assuming 100% adherence rates.

|  | **Strategy** | **NS** | **FS** | **COL** | **FIT** | **HS-gFOBT** | **FIT-DNA** | **CTC** | **SEPT9 (1Y)** | **SEPT9 (2Y)** | **SEPT9 (3Y)** |
| --- | --- | --- | --- | --- | --- | --- | --- | --- | --- | --- | --- |
| CRC (per 1000 person- year) | All | 2.05 | 1.02 | 0.57 | 0.59 | 0.59 | 0.67 | 0.79 | 0.61 | 0.77 | 0.92 |
|  | Distal | 1.40 | 0.49 | 0.38 | 0.39 | 0.40 | 0.45 | 0.53 | 0.41 | 0.51 | 0.61 |
|  | Proximal | 0.65 | 0.53 | 0.19 | 0.20 | 0.20 | 0.22 | 0.26 | 0.21 | 0.26 | 0.30 |
| CRC Death (per 1000 person-year) | All | 0.94 | 0.35 | 0.13 | 0.13 | 0.13 | 0.16 | 0.22 | 0.14 | 0.19 | 0.24 |
|  | Distal | 0.64 | 0.13 | 0.09 | 0.08 | 0.09 | 0.11 | 0.14 | 0.09 | 0.12 | 0.16 |
|  | Proximal | 0.29 | 0.22 | 0.04 | 0.04 | 0.04 | 0.05 | 0.07 | 0.05 | 0.06 | 0.08 |
| LY (year) |  | 31.85 | 32.07 | 32.16 | 32.15 | 32.15 | 32.14 | 32.13 | 32.15 | 32.13 | 32.11 |
| LYG (year, per 1000 person screened) |  |  | 221 | 306 | 302 | 303 | 293 | 277 | 296 | 278 | 261 |
| CRC death averted (per 1000 person screened) |  |  | 19  (15 -22) | 26  (22-29) | 26  (22-29) | 26  (22-29) | 25  (21-28) | 23  (19-27) | 25  (22-29) | 24  (20-27) | 22  (18-26) |
| CRC averted (per 1000 person screened) |  |  | 32  (26-38) | 46  (40-51) | 45  (39-51) | 45  (39-50) | 42  (36-48) | 39  (33-44) | 44  (39-50) | 39  (34-45) | 35  (29-41) |
| AE (per 1000 person screened) |  |  | 2.2 | 7.6 | 4.2 | 5.1 | 4.1 | 6.1 | 6.5 | 5.5 | 4.8 |
| Number of colonoscopy and non-colonoscopy tests for each screening strategy |  |  |  |  |  |  |  |  |  |  |  |
|  | FS (per 1000 screened) |  | 4,397 | - | - | - | - | - | - | - | - |
|  | COL (per 1000 screened) |  | 871 | 3,777 | 2,095 | 2,559 | 2,048 | 1,623 | 3,286 | 2,799 | 2,400 |
|  | FIT (per 1000 screened) |  | - | - | 15,151 | - | - | - | - | - | - |
|  | HS-gFOBT (per 1000 screened) |  | - | - | - | 11,789 | - | - | - | - | - |
|  | FIT-DNA (per 1000 screened) |  | - | - | - | - | 5,687 | - | - | - | - |
|  | CTC (per 1000 screened) |  | - | - | - | - | - | 3,767 | - | - | - |
|  | SEPT9 (per 1000 screened) |  | - | - | - | - | - | - | 6,168 | 5,396 | 4,669 |

CRC: colorectal cancer; LY: life years; LYG: life years gained; AE: adverse events; NS: no screening; FS: flexible sigmoidoscopy every 5 years; COL: colonoscopy every 10 years; FIT: fecal immunochemical testing every year; HS-gFOBT: high-sensitivity guaiac-based fecal occult blood test every year; FIT-DNA: multitarget stool DNA testing every 3 years; CTC: computed tomographic colonography every 5 years; SEPT9 (1Y): SEPT9 DNA test every year; SEPT9 (2Y): SEPT9 DNA every two years; SEPT (3Y): SEPT9 DNA every three years.

**eTable 5.** Outcomes of screening strategies assuming reported adherence rates.

|  | **Strategy** | **NS** | **FS** | **COL** | **FIT** | **HS-gFOBT** | **FIT-DNA** | **CTC** | **SEPT9 (1Y)** | **SEPT9 (2Y)** | **SEPT9 (3Y)** |
| --- | --- | --- | --- | --- | --- | --- | --- | --- | --- | --- | --- |
| CRC (per 1000 person-year) | All | 2.05 | 1.44 | 0.94 | 1.38 | 1.54 | 1.24 | 1.53 | 0.84 | 0.97 | 1.07 |
|  | Distal | 1.40 | 0.85 | 0.63 | 0.93 | 1.04 | 0.83 | 1.04 | 0.56 | 0.65 | 0.72 |
|  | Proximal | 0.65 | 0.58 | 0.31 | 0.45 | 0.49 | 0.41 | 0.49 | 0.28 | 0.32 | 0.35 |
| CRC Death (per 1000 person-year) | All | 0.94 | 0.58 | 0.32 | 0.49 | 0.59 | 0.43 | 0.62 | 0.22 | 0.27 | 0.32 |
|  | Distal | 0.64 | 0.33 | 0.21 | 0.33 | 0.40 | 0.29 | 0.42 | 0.15 | 0.18 | 0.21 |
|  | Proximal | 0.29 | 0.25 | 0.10 | 0.16 | 0.19 | 0.14 | 0.20 | 0.07 | 0.09 | 0.10 |
| LY (year) |  | 31.85 | 31.98 | 32.07 | 32.01 | 31.99 | 32.04 | 31.97 | 32.12 | 32.10 | 32.08 |
| LYG (year, per 1000 person screened) |  |  | 125 | 218 | 159 | 137 | 185 | 116 | 267 | 248 | 228 |
| CRC death averted (per 1000 person screened) |  |  | 11  (9-14) | 20  (16-24) | 14  911-17) | 11  (9-13) | 16  (13-19) | 10  (8-12) | 23  (18-27) | 21  (17-25) | 20  (16-24) |
| CRC averted (per 1000 person screened) |  |  | 19  (15-22) | 34  (27-41) | 20  (16-25) | 16  (13-19) | 25  (20-30) | 16  (13-19) | 37  (30-45) | 33  (26-40) | 30  (24-36) |
| AE (per 1000 person screened) |  |  | 0.6 | 3.7 | 0.8 | 0.8 | 1.1 | 1.5 | 3.4 | 2.8 | 2.5 |
| Number of colonoscopy and non-colonoscopy tests for each screening strategy |  |  |  |  |  |  |  |  |  |  |  |
|  | FS (per 1000 screened) |  | 1,897 | - | - | - | - | - | - | - | - |
|  | COL (per 1000 screened) |  | 199 | 1,858 | 379 | 377 | 568 | 305 | 1,708 | 1,443 | 1,243 |
|  | FIT (per 1000 screened) |  | - | - | 3,488 | - | - | - | - | - | - |
|  | HS-gFOBT (per 1000 screened) |  | - | - | - | 2,434 | - | - | - | - | - |
|  | FIT-DNA (per 1000 screened) |  | - | - | - | - | 2,517 | - | - | - | - |
|  | CTC (per 1000 screened) |  | - | - | - | - | - | 1,160 | - | - | - |
|  | SEPT9 (per 1000 screened) |  | - | - | - | - | - | - | 5,533 | 4,787 | 4,148 |

CRC: colorectal cancer; LY: life years; LYG: life years gained; AE: adverse events; NS: No screening; FS: flexible sigmoidoscopy every 5 years; COL: colonoscopy every 10 years; FIT: fecal immunochemical testing every year; HS-gFOBT: high-sensitivity guaiac-based fecal occult blood test every year; FIT-DNA: multitarget stool DNA testing every 3 years; CTC: computed tomographic colonography every 5 years; SEPT9 (1Y): SEPT9 DNA test every year; SEPT9 (2Y): SEPT9 DNA every two years; SEPT (3Y): SEPT9 DNA every three years.

**eFigure 1. A.** Structure of the FIT, HS-gFOBT, FIT-DNA, and SEPT9 screening strategies included in the microsimulation model.


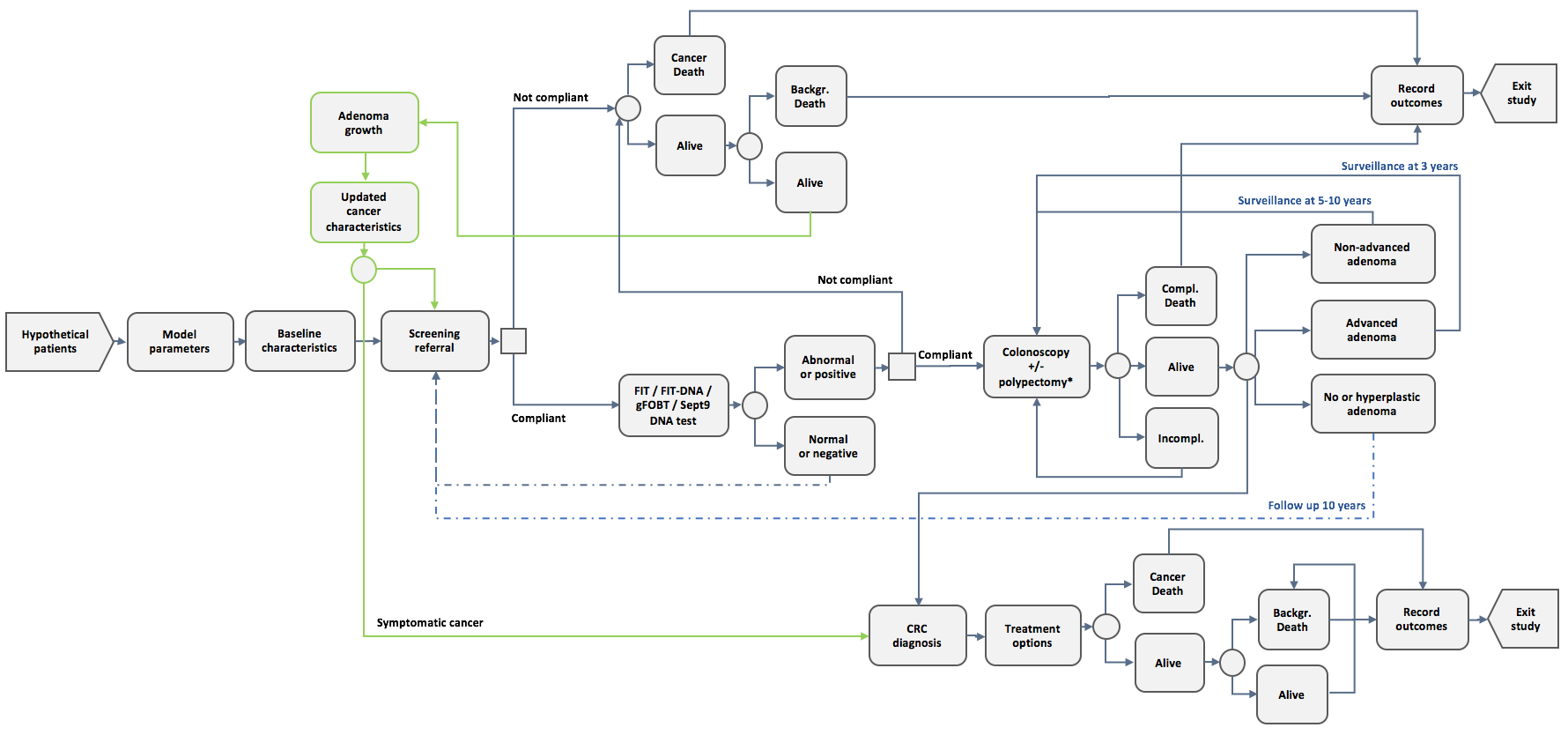


The diagram shows the structure of the model for four screening strategies: FIT, HS-gFOBT, FIT-DNA, and SEPT9. These are presented in a single diagram because they have identical structure but different values for model parameters and time intervals of screening referrals. The green lines indicate the time between two subsequent screening referrals in which non-compliant patients can develop adenoma and/or cancer (FIT and HS-gFOBT: 1 year; FIT-DNA: 3 years; SEPT9: 1, 2 or 3 years). The dotted lines indicate the time to follow-up. When the time on the dotted lines is not specified in the diagrams, it corresponds to the time interval between two screening referrals of a specific strategy.

FIT, fecal immunochemical testing every year; HS-gFOBT, high-sensitivity guaiac-based fecal occult blood test every year; FIT-DNA, multitarget stool DNA testing every 3 years; SEPT9, SEPT9 DNA test with intervals of 1, 2 or 3 years.

**eFigure 1.B.** Structure of the CT colonography screening strategy included in the microsimulation model.


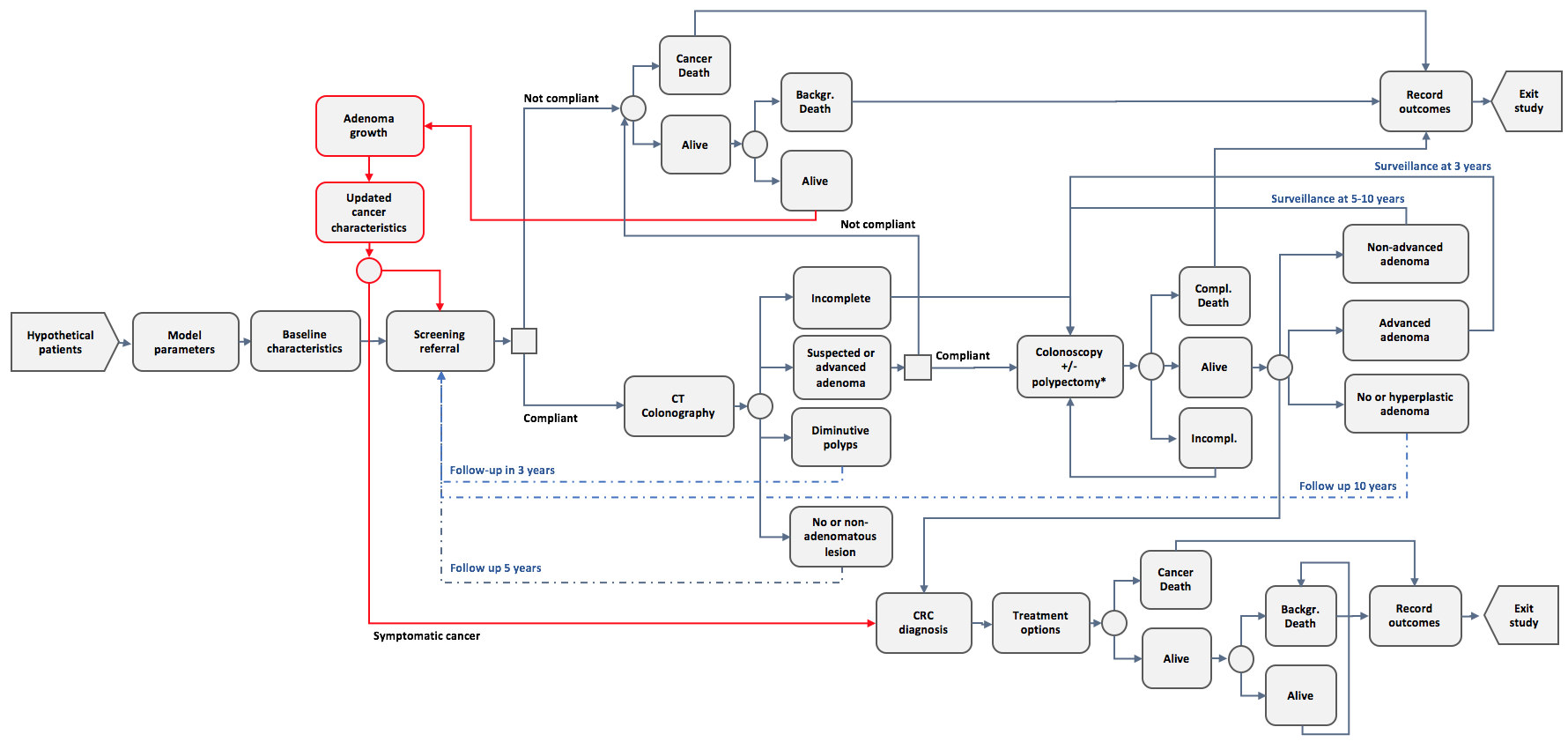


The diagram shows the structure of the model for CT colonography every 5 years. The red lines indicate the time between two subsequent screening referrals in which non-compliant patients can develop adenoma and/or cancer (CTC: 5 years). The dotted lines indicate the time to follow-up. When the time on the dotted lines is not specified in the diagrams, it corresponds to the time interval between two screening referrals of a specific strategy.

CTC computed tomographic colonography every 5 years.

**eFigure 1.C.** Structure of the flexible sigmoidoscopy screening strategy included in the microsimulation model.


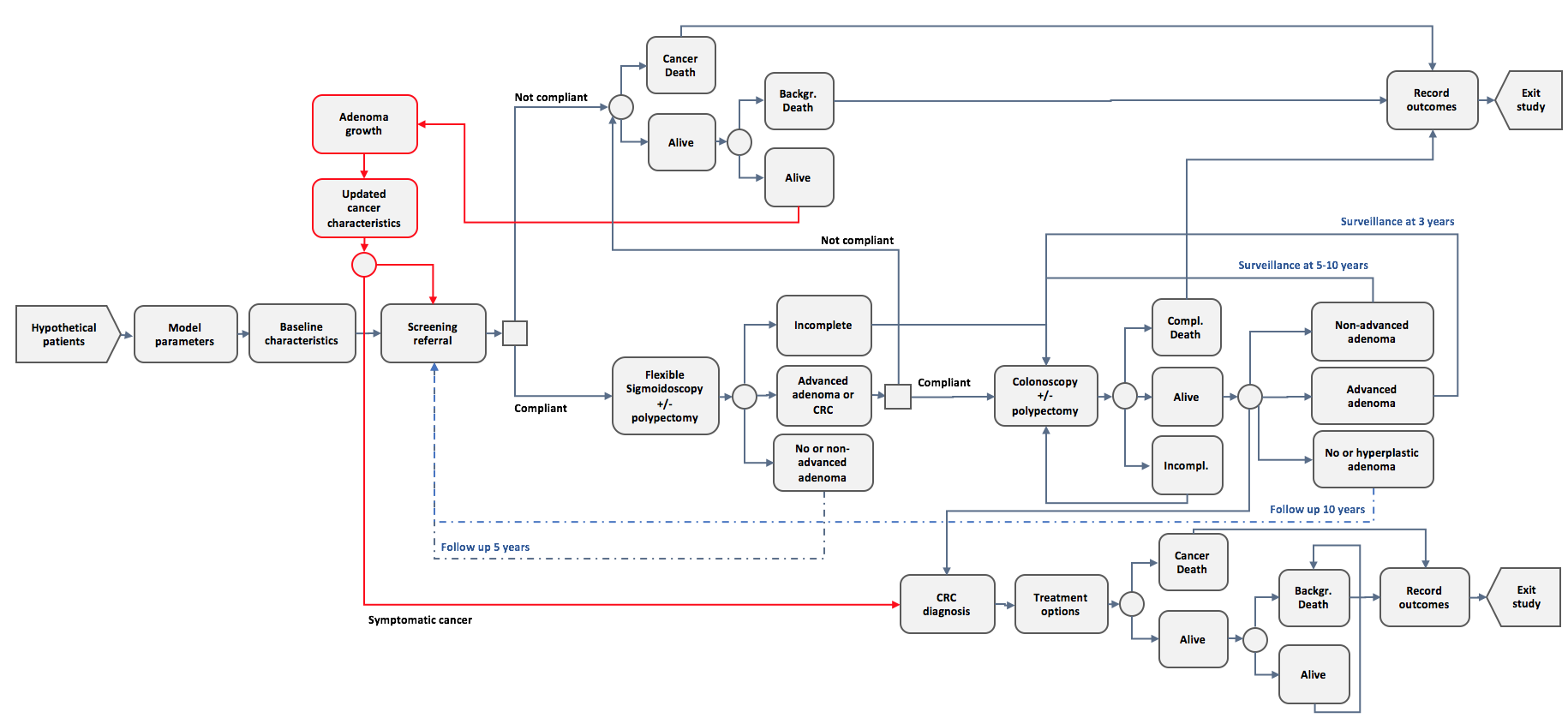


The diagrams show the structure of the model for flexible sigmoidoscopy every 5 years. The red lines in the models indicate the time between two subsequent screening referrals in which non-compliant patients can develop adenoma and/or cancer (FS: 5 years). The dotted lines indicate the time to follow-up. When the time on the dotted lines is not specified in the diagrams, it corresponds to the time interval between two screening referrals of a specific strategy.

FS, flexible sigmoidoscopy every 5 years.

**eFigure 1.D.** Modeling natural **history of CRC in the** microsimulation model.

The diagram shows the transition pathways between different stages of disease in our model. Definition of non-advanced adenoma, advanced adenoma, and pre-clinical cancer are based on the definition used by Brenner at al 2015.

**eFigure 2.** Model validation results: estimated age-specific CRC cases and CRC deaths

**eFigure 3**. The total number of non-colonoscopy and colonoscopy tests performed over time under the full adherence and reported adherence scenarios for each screening strategy.

1. Colonoscopy (every 10 years)

Number of tests: number of non-colonoscopy tests per 1,000 individuals

1. Flexible sigmoidoscopy (every 5 years)

Number of tests: number of non-colonoscopy tests per 1,000 individuals

Number of colonoscopies: number of colonoscopy tests per 1,000 individuals

1. FIT (every year)

Number of tests: number of non-colonoscopy tests per 1,000 individuals

Number of colonoscopies: number of colonoscopy tests per 1,000 individuals

1. HS-gFOBT (every year)

Number of tests: number of non-colonoscopy tests per 1,000 individuals

Number of colonoscopies: number of colonoscopy tests per 1,000 individuals

1. FIT-DNA (every 3 years)

Number of tests: number of non-colonoscopy tests per 1,000 individuals

Number of colonoscopies: number of colonoscopy tests per 1,000 individuals

1. CTC (every 5 years)

Number of tests: number of non-colonoscopy tests per 1,000 individuals

Number of colonoscopies: number of colonoscopy tests per 1,000 individuals

1. SEPT9 DNA (every 1 year).

Number of tests: number of non-colonoscopy tests per 1,000 individuals

Number of colonoscopies: number of colonoscopy tests per 1,000 individuals

1. SEPT9 DNA (every 2 years)

Number of tests: number of non-colonoscopy tests per 1,000 individuals

Number of colonoscopies: number of colonoscopy tests per 1,000 individuals

1. SEPT9 DNA (every 3 years)

Number of tests: number of non-colonoscopy tests per 1,000 individuals

Number of colonoscopies: number of colonoscopy tests per 1,000 individuals

**eFigure 4**. Colorectal cancer cases and deaths averted across the screening strategies for different adherence rates. The horizontal red line presents the outcomes for colonoscopy every ten years assuming reported adherence rate (38%).

1. CRC cases averted

1. CRC deaths averted

1. Number of tests performed

**eReferences**

1. Brenner H, Altenhofen L, Stock C, Hoffmeister M. Prevention, early detection, and overdiagnosis of colorectal cancer within 10 years of screening colonoscopy in Germany. Clinical Gastroenterology and Hepatology 2015;13:717-23.

2. Schoenfeld P, Cash B, Flood A, et al. Colonoscopic screening of average-risk women for colorectal neoplasia. New England Journal of Medicine 2005;352:2061-8.

3. Atkin WS, Edwards R, Kralj-Hans I, et al. Once-only flexible sigmoidoscopy screening in prevention of colorectal cancer: a multicentre randomised controlled trial. The Lancet 2010;375:1624-33.

4. Schoen RE, Pinsky PF, Weissfeld JL, et al. Colorectal-cancer incidence and mortality with screening flexible sigmoidoscopy. New England Journal of Medicine 2012;366:2345-57.

5. O’Connell JB, Maggard MA, Ko CY. Colon cancer survival rates with the new American Joint Committee on Cancer sixth edition staging. Journal of the National Cancer Institute 2004;96:1420-5.

6. Rutter CM, Knudsen AB, Marsh TL, et al. Validation of models used to inform colorectal cancer screening guidelines: accuracy and implications. Medical Decision Making 2016;36:604-14.

7. Brenner H, Altenhofen L, Katalinic A, Lansdorp-Vogelaar I, Hoffmeister M. Sojourn time of preclinical colorectal cancer by sex and age: estimates from the German national screening colonoscopy database. American journal of epidemiology 2011;174:1140-6.

8. Brenner H, Hoffmeister M, Stegmaier C, Brenner G, Altenhofen L, Haug U. Risk of progression of advanced adenomas to colorectal cancer by age and sex: estimates based on 840,149 screening colonoscopies. Gut 2007.

9. Kuntz KM, Lansdorp-Vogelaar I, Rutter CM, et al. A systematic comparison of microsimulation models of colorectal cancer: the role of assumptions about adenoma progression. Medical Decision Making 2011;31:530-9.

10. Knudsen AB, Zauber AG, Rutter CM, et al. Estimation of benefits, burden, and harms of colorectal cancer screening strategies: modeling study for the US Preventive Services Task Force. Jama 2016;315:2595-609.
